# Correction: On escape criterion of an orbit with *s*−convexity and illustrations of the behavior shifts in Mandelbrot and Julia set fractals

**DOI:** 10.1371/journal.pone.0320234

**Published:** 2025-03-10

**Authors:** The PLOS One Staff

## Notice of republication

This article was republished on February 14, 2025, to correct an error in the funding statement that was introduced during the typesetting process. The publisher apologizes for this error. Please download this article again to view the correct version. The originally published, uncorrected article and the republished, corrected articles are provided here for reference.

## Supporting information

S1 File Originally published, uncorrected article. (PDF)

S2 File Republished, corrected article. (PDF)

## References

[1] Alam KH, Rohen Y, Saleem N, Aphane M, Razzaque A. On escape criterion of an orbit with s-convexity and illustrations of the behavior shifts in Mandelbrot and Julia set fractals. PLoS One. 2025;20(1):e0312197. doi: 10.1371/journal.pone.0312197 39775582 PMC11706479

