# Supplementary figures and images for: On escape criterion of an orbit with s−convexity and illustrations of the behavior shifts in Mandelbrot and Julia set fractals
Source: PLoS One. 2025 Jan 7;20(1):e0312197. doi: 10.1371/journal.pone.0312197 (PMC11706479; doi:10.1371/journal.pone.0312197)

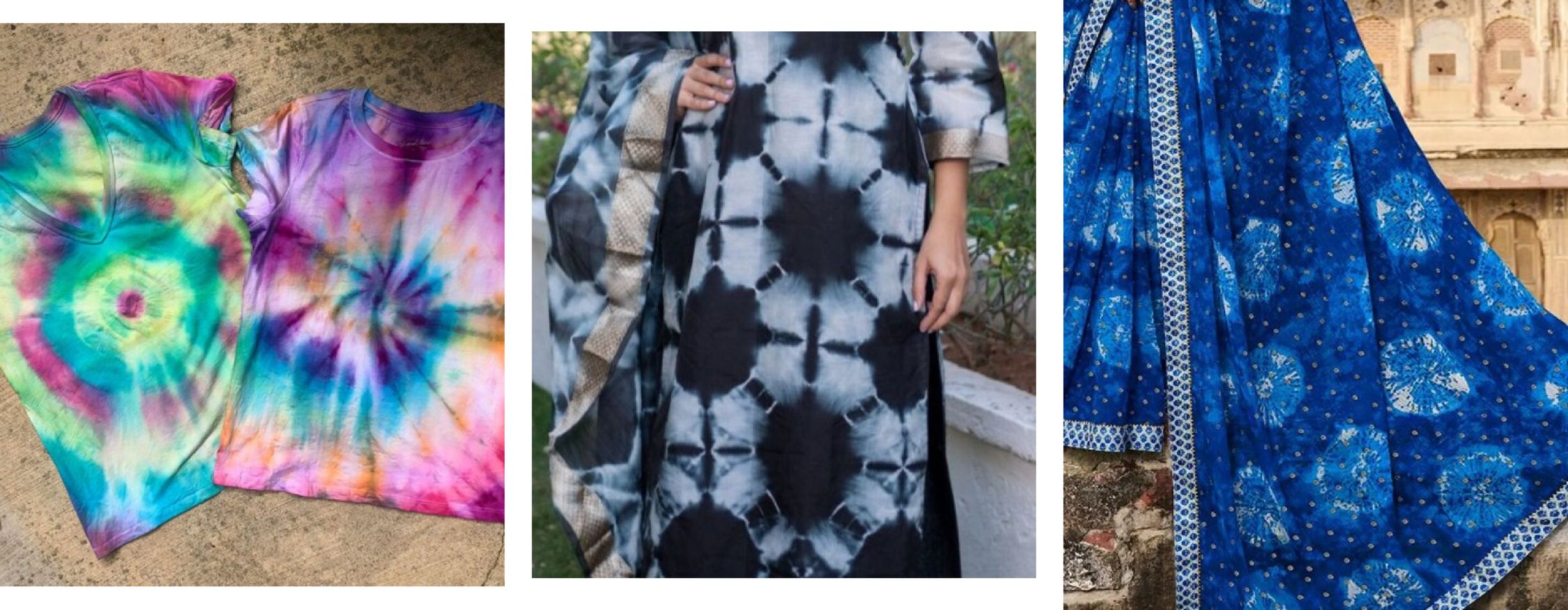

Supplement: S1 Fig — (TIF) [file pone.0312197.s001.tif]

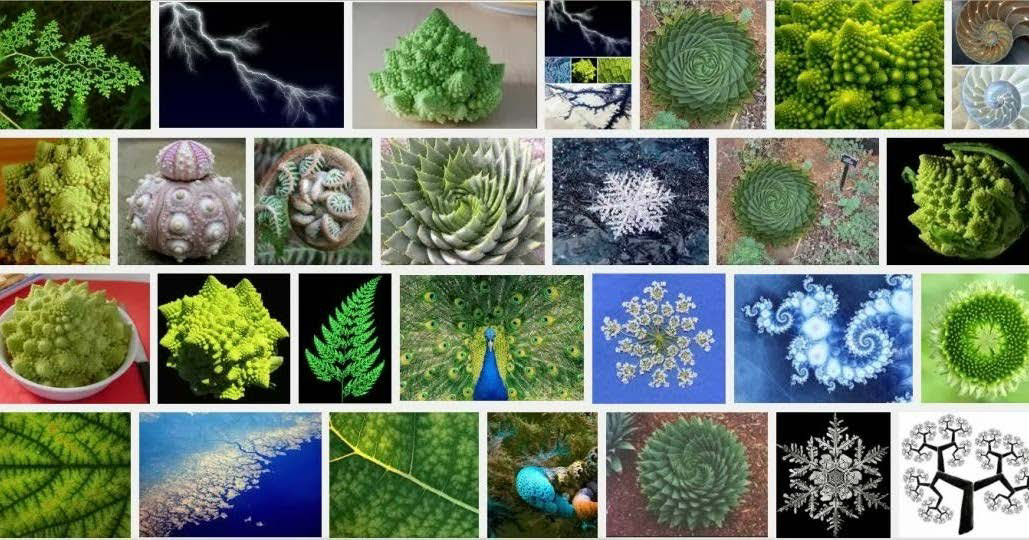

Supplement: S2 Fig — (TIF) [file pone.0312197.s002.tif]

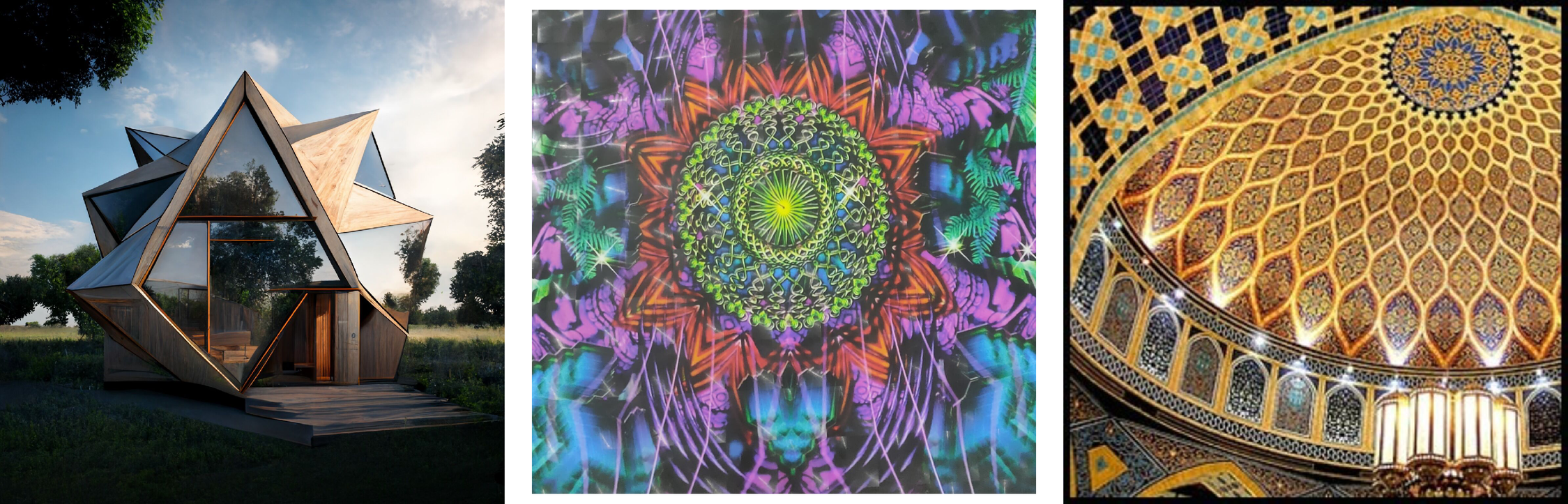

Supplement: S3 Fig — (TIF) [file pone.0312197.s003.tif]

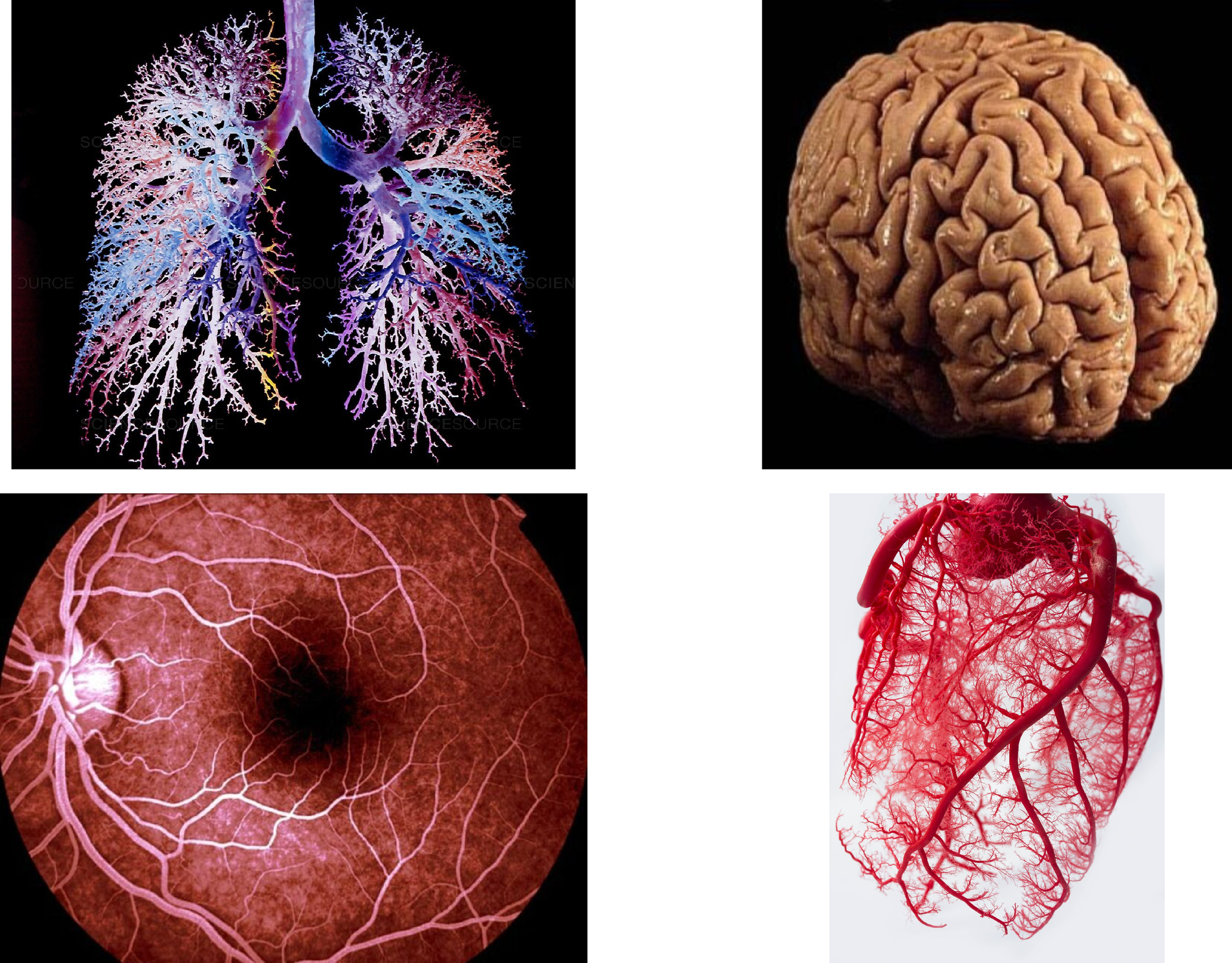

Supplement: S4 Fig — (TIF) [file pone.0312197.s004.tif]

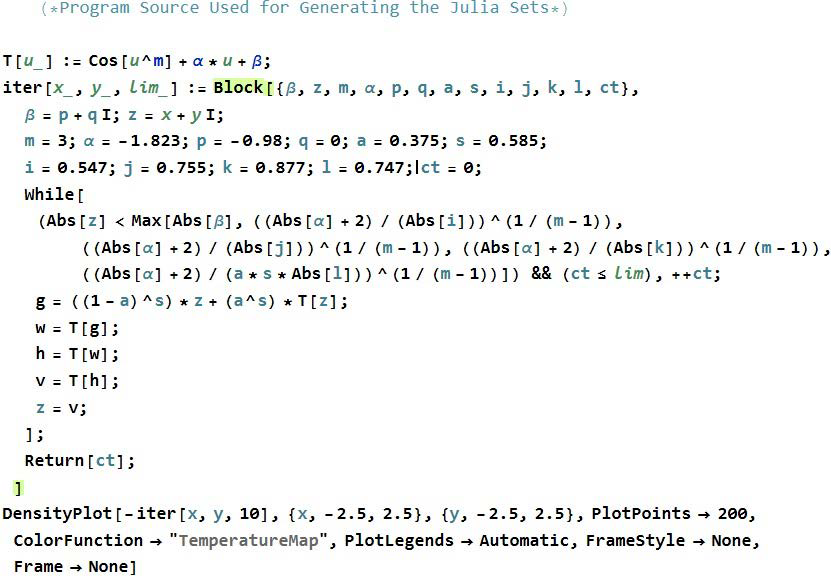

Supplement: S5 Fig — (TIF) [file pone.0312197.s005.tif]

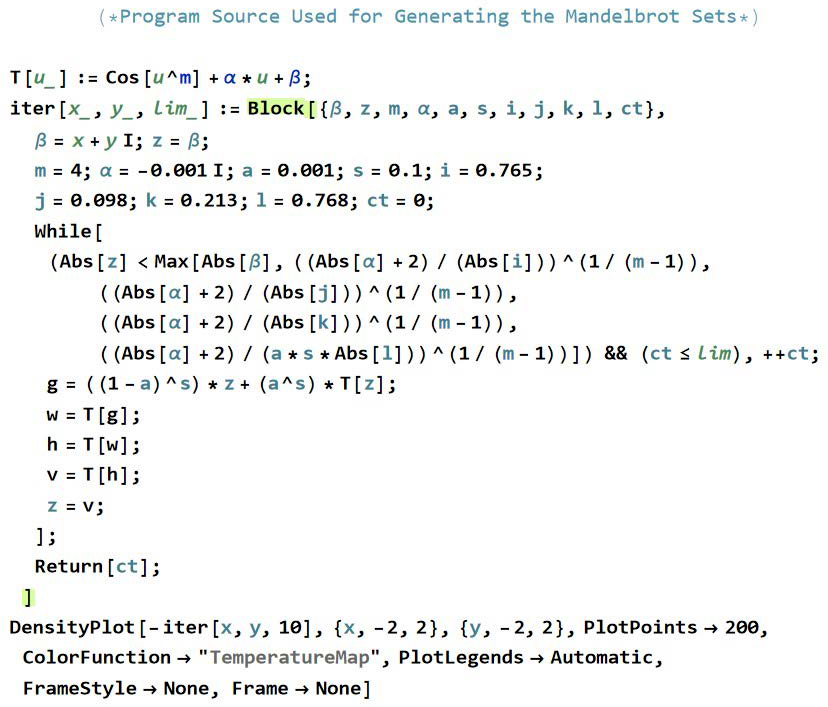

Supplement: S6 Fig — (TIF) [file pone.0312197.s006.tif]
