# Supplementary material for: Correction: On escape criterion of an orbit with s−convexity and illustrations of the behavior shifts in Mandelbrot and Julia set fractals
Source: PLoS One. 2025 Mar 10;20(3):e0320234. doi: 10.1371/journal.pone.0320234 (PMC11892803; doi:10.1371/journal.pone.0320234)
Supplement: S2 File — (PDF) [file pone.0320234.s002.pdf]

PLOS ONE

Check for updates

RESEARCH ARTICLE

On escape criterion of an orbit with s−convexity and illustrations of the behavior shifts in Mandelbrot and Julia set fractals

Khairul Habib Alam[1]*, Yumnam Rohen[1,2], Naeem Saleem[3,4]*, Maggie Aphane[4], Asima Razzaque[5,6]

1 Department of Mathematics, National Institute of Technology Manipur, Imphal, Manipur, India, **2** Department of Mathematics, Manipur University, Imphal, Manipur, India, **3** Department of Mathematics, University of Management and Technology, Lahore, Pakistan, **4** Department of Mathematics and Applied Mathematics, Sefako Makgatho Health Sciences University, Pretoria, South Africa, **5** Department of Basic Sciences, Preparatory Year, King Faisal University, Al-Ahsa, Saudi Arabia, **6** Department of Mathematics, College of Science, King Faisal University, Al-Ahsa, Saudi Arabia

* alamkh@nitmanipur.ac.in (KHA); naeem.saleem2@gmail.com (NS)

OPEN ACCESS

Citation: Alam KH, Rohen Y, Saleem N, Aphane M, Razzaque A (2025) On escape criterion of an orbit with s−convexity and illustrations of the behavior shifts in Mandelbrot and Julia set fractals. PLoS ONE 20(1): e0312197. https://doi.org/10.1371/journal.pone.0312197

Editor: Rizwan Anjum, University of Education, PAKISTAN

Received: August 26, 2024

Accepted: October 2, 2024

Published: January 7, 2025

Copyright: © 2025 Alam et al. This is an open access article distributed under the terms of the Creative Commons Attribution License, which permits unrestricted use, distribution, and reproduction in any medium, provided the original author and source are credited.

Data Availability Statement: All relevant data are within the paper and its Supporting information files.

Funding: This work was supported by the Deanship of Scientific Research, Vice Presidency for Graduate Studies and Scientific Research, King Faisal University, Saudi Arabia [KFU242044].

Competing interests: The authors have declared that no competing interests exist.

Abstract

Our study presents a novel orbit with s−convexity, for illustration of the behavior shift in the fractals. We provide a theorem to demonstrate the escape criterion for transcendental cosine functions of the type $T_{\alpha,\beta}(u) = \cos(u^m)+\alpha u + \beta$, for $u, \alpha, \beta \in \mathbb{C}$ and $m \geq 2$. We also demonstrate the impact of the parameters on the formatted fractals with numerical examples and graphical illustrations using the MATHEMATICA software, algorithm, and colormap. Moreover, we observe that the Julia set appears when we widen the Mandelbrot set at its petal edges, suggesting that each Mandelbrot set point contains a sizable quantity of Julia set picture data. It is commonly known that fractal geometry may capture the complexity of many intricate structures that exist in our surroundings.

1 Introduction

A branch of mathematics that is still developing is fixed point theory, which is connected to functional analysis and topology. Starting with the concept of proofing of the very famous Banach fixed point theorem [1] that uses the idea of approximations, many researchers developed the theory in different directions. Certain fixed point iteration procedures are frequently employed to estimate fixed points involving mappings which are linear contractions [2–4], rational contractions [5–7], hybrid contractions [8, 9], and many more. As an illustration, we can consider various iterations like Picard [10], Mann [11], Ishikawa [12], and Noor [13]. Recently, Alam et al. [14] studied an iteration process involving two mappings that exhibit weak compatibility and meet a generalized contractive condition. Additionally, in [15], they examined the Fibonacci-Ishikawa iteration for monotone asymptotically non-expansive mappings and applied it to approximate the solution of a Caputo-type nonlinear fractional differential equation. Also, in [16] applied efficient iteration to a practical problem by estimating solutions for a fractional Volterra-Fredholm integro-differential equation. In this context, we

are interested in the AI iteration procedure introduced by Ofem et al. [17] which results in a faster fixed point approximation in the literature.

In addition, several academics suggested applying s-convexity like the Jungck-Noor iteration with s–convexity [18], the Jungck-Mann and Jungck-Ishikawa iterations with s–convexity [19], the Ishikawa iteration with s–convexity [20], the Noor iteration with s–convexity [21], the SP–iteration with s–convexity [22], and the S–iteration with s–convexity [23] were proposed. There are two aspects to the study of these different iteration processes. First off, compared to normal iteration procedures, these different iteration processes achieve faster convergence. Second, every iteration exhibits unique dynamics and behavior that are intriguing from both an applications and graphics perspective (see [14, 15, 24–27]).

For many years, the study and investigation of fractals have been an important aspect of mathematics and computer science, and its impact has been witnessed in several domains [28], including art, physics [29], biology [30], and finance. Fractals are multifaceted geometric forms that show self-similarity at many scales, which implies that at any magnification, they appear to be the same or similar. The time frame 'Fractals Era' began at the end of the 20th century when advances in computational graphics and processing capacity contributed to the increased interest in the study of fractals. The following are certain significant characteristics and advancements during the Fractals Era.

- Mandelbrot Set: Introduction of the Mandelbrot Set [31, 32] by mathematician Benoit B. Mandelbrot in 1979 marked a major turning point in the development of the notion of fractals. Applying a basic mathematical technique to complex numbers repeatedly yields one of the most well-known fractals: the Mandelbrot Set.
- Julia Set: Another well-known fractal that is closely connected to the Mandelbrot Set is the Julia Set [33, 34]. Gaston Julia, a French mathematician investigated them in the early 20th century. A similar iterative procedure is used to build the Julia Set, but instead of changing the complex parameter as in the Mandelbrot Set, it is fixed.
- Computer graphics: Proficiency in computers has made it possible for scholars and individuals to create and display fractals with more intricacy and detail. People could now explore and produce their fractal pictures and animations [25] by using fractal-generating software (Mathematica, Matlab, Apophysis, etc.), which gained popularity.
- Chaotic Dynamics: Complex systems that demonstrate sensitive dependency on beginning conditions are the subject of chaotic dynamics, a branch of research strongly related to fractals. Studying fractals has helped us comprehend chaos theory better since they frequently arise from chaotic systems [24].
- Applications: Fractals have been used in signal processing [35], data compression, picture compression [36], video compression [37], and modelling of mountain ranges, water distribution networks [38], clouds, human body organs [39], and other natural phenomena. Architecture and urban planning [33] have also made use of fractal geometry.
- Popularization and Art: In addition to scientists and mathematicians, fractals captivated the interest of fans and artists. As a result of artists producing complex and captivating works of art [25] based on fractal patterns, fractal art emerged as a distinct genre.
- Mathematical Study: Researchers are still delving into the theoretical elements of fractals [21, 40, 41], finding previously undiscovered characteristics and relationships. Numerous mathematical fields, including geometry, dynamical systems [27], and topology, are related to the study of fractals.

The Fractals Era has influenced our comprehension of mathematical and natural events and stimulated creativity in a wide range of fields.

The first and most obvious generalization of the Mandelbrot set is using the function $u^m + \beta$ in place of the second-degree polynomial [42, 43]. The literature also looked at functions that fall under various categories [22, 42–45]. The study of Mandelbrot sets has also been extended from complex number systems to octonions [46], bicomplex numbers, quaternions, and so on. Several cyclical techniques, such as inversion fractals, ν−variable fractals, superfractals [47], and biomorphs [48], are used to locate fixed points in an identifiable map in order to create fractals utilizing fixed-point theory. Rani et al. [49] visualized Julia and Mandelbrot sets using the Mann iteration. Afterward, Julia and Mandelbrot sets were visualized in [50] using the Ishikawa iteration. Li et al. used the Jungck-Mann iteration procedure in [51]. Various scholars employed distinct iterative techniques, as exemplified by the Jungck-CR iterative formulas with a particular convexity [52]. Similar to this, an S-iteration procedure with s-convexity was employed in [23]. Next, s-convexity, Jungck-Mann, and Jungck-Ishikawa iterations were applied in [19, 53]. In [21], s-convexity and the Noor orbit were employed.

Inspired by the aforementioned, we present the s−convex AI iteration and use it to produce fractals for a transcendental cosine function. For the given function and the resultant orbit with convexity condition, we give a result to illustrate the escape criterion. Furthermore, we investigate the influence of the included factors on the chaotic behavior of generated fractals and use MATHEMATICA to provide numerical and graphical examples of the generated complex fractals. It is commonly known that fractal geometry may capture the complexity of many intricate structures that exist in our surroundings. In practice, fractals can represent structures and surfaces that traditional Euclidean geometry is difficult to describe. Generated fractals, pivotal in fabric design (e.g., batik, kalamkari), revolutionized the industry by automating processes, facilitating scalable designs, and minimizing errors. This fosters global collaboration, reduces costs, and promotes sustainability, driving market growth.

The manuscript consists of five sections. In section 1, we have literature on the context. In section 2, we will discuss some basic definitions and a discussion on some related useful terms. In section 3, we will prove the criterion theorem for escaping the orbit. Section 4 will have two subsections describing the illustrations of the behavior shifts in the Mandelbrot and Julia set fractals respectively, followed by conclusions in section 5.

2 Preliminaries

This section introduces some basic definitions and a discussion on some related useful terms. In the complex plane $\mathbb{C}$, let T: $C \to C$ be any self mapping. Then, the AI iteration procedure [17] is described as

$$\begin{cases} u_{n+1} = Tv_n \\ v_n = Tw_n \\ w_n = Tx_n \\ x_n = aTu_n + (1-a)u_n, \quad n \in \mathbb{N}. \end{cases}$$

for any choice $u_0 \in \mathbb{C}$, where $a \in (0, 1]$.

Definition 2.1. [33, 34] *A collection of complex numbers such that the orbits do not converge to an infinite point is a filled Julia set. If $T : \mathbb{C} \to \mathbb{C}$ is a polynomial of degree $m(\geq 2)$, then the boundary set ∂F_T of the set $F_T = \{u \in \mathbb{C} : \{|Tu_n|\} \text{ is bounded}\}$ is known as the Julia set.*

Definition 2.2. [31, 32] *All of the parameter values* β *for which the filled-in Julia set of* $T(u) = u^2 + \beta$ *is connected to comprise the Mandelbrot set M. That is,* $M = \{u \in \mathbb{C} : \partial F_T \text{ is connected}\}$ *or* $M = \{u \in \mathbb{C} : \{|Tu_n|\} \nrightarrow \infty \text{ whenever } n \to \infty\}$.

There are several generalizations of the convex combination in the literature, s–convex combination is one example of such generalizations.

Definition 2.3. [54] *For a finite set of complex numbers* $u_1, u_2, \ldots, u_n \in \mathbb{C}$, *the s–convex combination is presented as* $a_1^s u_1 + a_2^s u_2 + \cdots + a_n^s u_n$, *where* $0 \le a_i \le 1$ *for all* $i \in \{1, 2, \ldots, n\}$ *so that* $\sum_{i=1}^{n} a_i = 1$.

Let us observe that, for $s = 1$, the s–convex combination simplifies to the conventional convex combination.

3 Escape criterion

The general escape criterion of the AI orbit with s–convex combination connected to transcendental cosine functions in the complex plane is examined in this section. In the AI iteration, we now substitute the concept of s–convex combination to get the AI orbit with s–convexity.

Definition 3.1. *In the complex plane* $\mathbb{C}$, *let* $T : \mathbb{C} \to \mathbb{C}$ *be any self mapping. Then, the AI orbit with an s–convexity is described as*

$$\begin{cases} u_{n+1} = Tv_n \\ v_n = Tw_n \\ w_n = Tx_n \\ x_n = a^s Tu_n + (1-a)^s u_n, \ \forall n \in \mathbb{N} \cup \{0\}, \end{cases} \tag{1}$$

for any choice $u_0 \in \mathbb{C}$, *where* $a, s \in (0, 1]$.

For the function $\cos(u^m)$, we know that $|\cos(u^m)| \le 1$ and consequently

$$|\cos(u^m)| = \left|1 - \frac{u^{2m}}{2!} + \frac{u^{4m}}{4!} - \cdots\right| \ge |\gamma||u^m|,$$

for some $0 < |\gamma| \le 1$ and for all $u \in \mathbb{C}$ but for which $|\gamma| = 0$.

The following serves as the escape criterion for the orbit defined in (1).

Theorem 3.1. *Let us consider the transcendental complex cosine function* $T_{\alpha,\beta}(u) = \cos(u^m) + \alpha u + \beta$, *for all* $u \in \mathbb{C}$, *where* $\alpha, \beta \in \mathbb{C}$ *and* $m \ge 2$. *Then the AI orbit* $\{u_n\}$ *with s–convexity is so that* $|u_n| \to \infty$ *whenever* $n \to \infty$, *if*

$$|u| \ge |\beta| \ge \left(\frac{|\alpha| + 2}{|\gamma_1|}\right)^{\frac{1}{m-1}}, |u| \ge |\beta| \ge \left(\frac{|\alpha| + 2}{|\gamma_2|}\right)^{\frac{1}{m-1}},$$

$$|u| \ge |\beta| \ge \left(\frac{|\alpha| + 2}{|\gamma_3|}\right)^{\frac{1}{m-1}} \text{ and } |u| \ge |\beta| \ge \left(\frac{|\alpha| + 2}{as|\gamma_4|}\right)^{\frac{1}{m-1}}.$$

Proof. For $n = 0$, let $u_0 = u$. Then from the AI iteration procedure with s−convexity, we have

$$\begin{aligned} |x_0| &= |a^s T u_0 + (1-a)^s u_0| \\ &= |a^s T u + (1-a)^s u| \\ &= |a^s[\cos(u^m) + \alpha u + \beta] + (1-a)^s u| \\ &\geq |a^s|[|\cos(u^m)| - |\alpha u| - |\beta|] - |(1-a)^s u|. \end{aligned}$$

Now, there exists $\gamma_4 \in \mathbb{C}$ with $|\gamma_4| \in (0, 1]$ so that $|\cos(u^m)| \geq |\gamma_4||u^m|$, for all $u \in \mathbb{C}$ but for which $|\gamma_4| = 0$. Also, $a, s \in (0, 1]$ implies $a^s \geq as$ and from the binomial expansion of $(1-a)^s$, we have $(1-a)^s \leq 1-as$. Hence utilizing $|u| \geq |\beta|$, we get

$$\begin{aligned} |x_0| &\geq as[|\gamma_4||u^m| - |\alpha u| - |u|] - |(1-as)||u| \\ &\geq as[|\gamma_4||u^m| - |\alpha||u| - |u|] - |(1-as)||u| \\ &= as[|\gamma_4||u^m| - |\alpha||u| - |u|] - |u| + as|u| \\ &= as[|\gamma_4||u^m| - |\alpha||u|] - |u| \\ &= as|\gamma_4||u^m| - |u|[as|\alpha| + 1] \\ &\geq as|\gamma_4||u^m| - |u|[|\alpha| + 1], \text{ since } as < 1 \\ &= |u|(as|\gamma_4||u^{m-1}| - (|\alpha| + 1)). \end{aligned}$$

Since $|u| \geq \left(\frac{|\alpha|+2}{as|\gamma_4|}\right)^{\frac{1}{m-1}}$, we have $|x_0| \geq |u| \geq |\beta|$.

This brings us to the next iteration of the AI procedure for $x_0 = x$

$$\begin{aligned} |w_0| &= |Tx_0| \\ &= |Tx| \\ &= |\cos(x^m) + \alpha x + \beta| \\ &\geq |\cos(x^m)| - |\alpha||x| - |\beta|. \end{aligned}$$

Now, there exists $\gamma_3 \in \mathbb{C}$ with $|\gamma_3| \in (0, 1]$ so that $|\cos(x^m)| \geq |\gamma_3||x^m|$, for all $x \in \mathbb{C}$ but for which $|\gamma_3| = 0$. Hence utilizing $|x| \geq |u| \geq |\beta|$, we get

$$\begin{aligned} |w_0| &\geq |\gamma_3||x^m| - |\alpha x| - |x| \\ &= |x|(|\gamma_3||x^{m-1}| - (|\alpha| + 1)). \end{aligned}$$

Since $|x| \geq \left(\frac{|\alpha|+2}{|\gamma_3|}\right)^{\frac{1}{m-1}}$, we have $|w_0| \geq |x| \geq |u| \geq |\beta|$.

This brings us to the next iteration of the AI procedure for $w_0 = w$

$$\begin{aligned} |v_0| &= |Tw_0| \\ &= |Tw| \\ &= |\cos(w^m) + \alpha w + \beta| \\ &\geq |\cos(w^m)| - |\alpha||w| - |\beta|. \end{aligned}$$

Now, there exists $\gamma_2 \in \mathbb{C}$ with $|\gamma_2| \in (0, 1]$ so that $|\cos(w^m)| \geq |\gamma_2||w^m|$, for all $w \in \mathbb{C}$ but for which $|\gamma_2| = 0$. Hence utilizing $|w| \geq |x| \geq |u| \geq |\beta|$, we get

$$\begin{aligned} |v_0| &\geq |\gamma_2||w^m| - |\alpha w| - |w| \\ &= |w|(|\gamma_3||w^{m-1}| - (|\alpha| + 1)). \end{aligned}$$

Since $|w| \geq \left(\frac{|\alpha|+2}{|\gamma_2|}\right)^{\frac{1}{m-1}}$, we have $|v_0| \geq |w| \geq |x| \geq |u| \geq |\beta|$.

This brings us to the next iteration of the AI procedure for $v_0 = v$

$$\begin{aligned} |u_1| &= |Tv_0| \\ &= |Tv| \\ &= |\cos(v^m) + \alpha v + \beta| \\ &\geq |\cos(v^m)| - |\alpha||v| - |\beta|. \end{aligned}$$

Now, there exists $\gamma_1 \in \mathbb{C}$ with $|\gamma_1| \in (0, 1]$ so that $|\cos(v^m)| \geq |\gamma_1||v^m|$, for all $v \in \mathbb{C}$ but for which $|\gamma_1| = 0$. Hence utilizing $|v| \geq |w| \geq |x| \geq |u| \geq |\beta|$, we get

$$\begin{aligned} |u_1| &\geq |\gamma_1||v^m| - |\alpha v| - |v| \\ &= |v|(|\gamma_1||v^{m-1}| - (|\alpha| + 1)) \\ &\geq |u|(|\gamma_1||v^{m-1}| - (|\alpha| + 1)). \end{aligned}$$

Consequently, for $n = 1$, we have

$$\begin{aligned} |u_2| &\geq |u_1|(|\gamma_1||v^{m-1}| - (|\alpha| + 1)) \\ &\geq |u|(|\gamma_1||v^{m-1}| - (|\alpha| + 1))^2. \end{aligned}$$

Continuing the iteration we have

$$\begin{aligned} |u_3| &\geq |u|(|\gamma_1||v^{m-1}| - (|\alpha| + 1))^3, \\ |u_4| &\geq |u|(|\gamma_1||v^{m-1}| - (|\alpha| + 1))^4, \\ &\vdots \\ |u_n| &\geq |u|(|\gamma_1||v^{m-1}| - (|\alpha| + 1))^n. \end{aligned}$$

Since $|u| \geq \left(\frac{|\alpha|+2}{|\gamma_1|}\right)^{\frac{1}{m-1}}$, we have $|u_n| \to \infty$ as $n \to \infty$.

Now we present subsequent corollaries that offer exploration methods for Julia and Mandelbrot sets.

Corollary 3.1. *Let us consider the transcendental complex cosine function* $T_{\alpha,\beta}(u) = \cos(u^m) + \alpha u + \beta$, *for all* $u \in \mathbb{C}$, *where* $\alpha, \beta \in \mathbb{C}$ *and* $m \geq 2$. *Then the AI orbit escapes to infinity, if*

$$|u| \geq |\beta| \geq \max\left\{ \left(\frac{|\alpha| + 2}{|\gamma_1|}\right)^{\frac{1}{m-1}}, \left(\frac{|\alpha| + 2}{|\gamma_2|}\right)^{\frac{1}{m-1}}, \left(\frac{|\alpha| + 2}{|\gamma_3|}\right)^{\frac{1}{m-1}}, \left(\frac{|\alpha| + 2}{as|\gamma_4|}\right)^{\frac{1}{m-1}} \right\}.$$

Corollary 3.2. Let us consider the transcendental complex cosine function $T_{\alpha,\beta}(u) = \cos(u^m) + \alpha u + \beta$, *for all* $u \in \mathbb{C}$, where $\alpha, \beta \in \mathbb{C}$ *and* $m \geq 2$. *Then the AI orbit escapes to infinity, if*

$$|u| \geq \max\left\{|\beta|, \left(\frac{|\alpha|+2}{|\gamma_1|}\right)^{\frac{1}{m-1}}, \left(\frac{|\alpha|+2}{|\gamma_2|}\right)^{\frac{1}{m-1}}, \left(\frac{|\alpha|+2}{|\gamma_3|}\right)^{\frac{1}{m-1}}, \left(\frac{|\alpha|+2}{as|\gamma_4|}\right)^{\frac{1}{m-1}}\right\}.$$

4 Generation of fractals

With MATHEMATICA 12.3, in this section, we try to obtain the non-classical chaotic fractals as Julia and Mandelbrot set in AI orbit with s−convexity within the area varying from [−0.1, 0.1]×[−0.1, 0.1] to [−5, 5]×[−5, 5]. A computer equipped with the following characteristics was used to evaluate the observations: 11th Gen Intel(R) Core(TM) i3–1115G4 @ 3.00GHz processor, 8 GB DDR3 RAM, and Microsoft Windows 11 Home Single Language (64-bit) operating system Version: 24H2, OS build: 26063.1, Feature Experience Pack 1000.26063.1.0.

Although fractal geometry and complex numbers are related to both Julia sets and Mandelbrot sets, they are also separate mathematical entities with some significant distinctions that are seen in the algorithms given in Tables 1 and 2 (MATHEMATICA source codes are in S5 and S6 Figs). The algorithm of Julia sets is usually examined in Table 1 by changing the beginning values of u_0 for a particular β to determine which points stay limited and which escape to infinity. Meanwhile, we have $u_0 = 0$ as the starting point/first iteration for the algorithm of Mandelbrot set in Table 2.

Fractals as Julia sets

This subsection illustrates the behavior shift in the fractals as Julia sets for the transcendental cosine function via the AI orbit with s−convexity. Additionally, the fractals alter significantly when even a small modification is made to any of the parameters. Consequently, we modify

Table 1. Algorithm for generation of fractals as Julia sets.

1.	Setup:
(i)	Define the transcendental cosine function $T_{\alpha,\beta}(u) = \cos(u^m) + \alpha u + \beta$.
(ii)	Consider a complex number $\beta = p + iq$
(iii)	Set the variables α, a, γ_1, γ_2, γ_3, γ_4, m, n, s, p, q to their initial values
(iv)	Take into account the initial iteration $u_0 = x + iy$
2.	Iterate:
	$u_{n+1} = Tv_n$
	$v_n = Tw_n$
	$w_n = Tx_n$
	$x_n = (1-a)^s u_n + a^s Tu_n$
3.	Stop:
	$\lvert u\rvert \geq \max\{\lvert\beta\rvert, \left(\frac{\lvert\alpha\rvert+2}{\lvert\gamma_1\rvert}\right)^{\frac{1}{m-1}}, \left(\frac{\lvert\alpha\rvert+2}{\lvert\gamma_2\rvert}\right)^{\frac{1}{m-1}}, \left(\frac{\lvert\alpha\rvert+2}{\lvert\gamma_3\rvert}\right)^{\frac{1}{m-1}}, \left(\frac{\lvert\alpha\rvert+2}{as\lvert\gamma_4\rvert}\right)^{\frac{1}{m-1}}\}$
4.	Count:
	The number of attempts made to escape.
5.	Colour:
	In accordance with the number of escape repetitions required.

https://doi.org/10.1371/journal.pone.0312197.t001

Table 2. Algorithm for generation of fractals as Mandelbrot sets.

1.	Setup:
(i)	Define the transcendental cosine function $T_{\alpha,\beta}(u) = \cos(u^m) + \alpha u + \beta$.
(ii)	Consider a complex number $\beta = x + iy$
(iii)	Set the variables α, a, γ_1, γ_2, γ_3, γ_4, m, n, s to their initial values
(iv)	Take into account $u = \beta$
2.	Iterate:
	$u_{n+1} = Tv_n$
	$v_n = Tw_n$
	$w_n = Tx_n$
	$x_n = (1-a)^s u_n + a^s Tu_n$
3.	Stop:
	$\lvert u\rvert \geq \max\{\lvert\beta\rvert, \left(\frac{\lvert\alpha\rvert+2}{\lvert\gamma_1\rvert}\right)^{\frac{1}{m-1}}, \left(\frac{\lvert\alpha\rvert+2}{\lvert\gamma_2\rvert}\right)^{\frac{1}{m-1}}, \left(\frac{\lvert\alpha\rvert+2}{\lvert\gamma_3\rvert}\right)^{\frac{1}{m-1}}, \left(\frac{\lvert\alpha\rvert+2}{as\lvert\gamma_4\rvert}\right)^{\frac{1}{m-1}}\}$
4.	Count:
	The number of attempts made to escape.
5.	Colour:
	In accordance with the number of escape repetitions required.

https://doi.org/10.1371/journal.pone.0312197.t002

nearly every parameter and generate the fractals for our orbit, which are shown in the images below (Reading direction: Row by row and Left to right).

The significant fractals that result from varying a parameter m (see Table 3) while holding the other parameters constant are shown in Fig 1. The numbers of chaotic attractors in fractals are increasing and the shape of fractals gets circular as the value of parameter m increases in number. The number of spokes in each Julia set is $2m$.

The fractals are made more beautiful by the parameter α. More aesthetically chaotic fractals can be observed in Fig 2 when the complex part of α decreases while maintaining the same values for the other parameters (as in Table 4). The color green vanished for positive complex value $0.9 + 0.8i$ of the parameter α.

The fractals in Fig 3 are made more dense by the parameter β in Table 5. The color red is displayed for real values of the parameter β, while yellow, green, and other colors are displayed for complex values of the parameter β. The chaos of color increases with an increase in the absolute value of β. However, there is no alteration in the fundamental shape.

There is a noticeable change in the fundamental shape with an increase in the value of the convexity parameter. However, there is no change in colors. Higher values add beauty to the

Table 3. Changes in parameter m for generating fractals as Julia set.

	m	α	β	a	s	γ_1	γ_2	γ_3	γ_4
(i)	2	$-0.004i$	$0.56 + 0.3i$	0.0238	0.7	0.087	0.0932	0.00405	0.6
(ii)	3	$-0.004i$	$0.56 + 0.3i$	0.0238	0.7	0.087	0.0932	0.00405	0.6
(iii)	4	$-0.004i$	$0.56 + 0.3i$	0.0238	0.7	0.087	0.0932	0.00405	0.6
(iv)	5	$-0.004i$	$0.56 + 0.3i$	0.0238	0.7	0.087	0.0932	0.00405	0.6
(v)	6	$-0.004i$	$0.56 + 0.3i$	0.0238	0.7	0.087	0.0932	0.00405	0.6
(vi)	7	$-0.004i$	$0.56 + 0.3i$	0.0238	0.7	0.087	0.0932	0.00405	0.6

https://doi.org/10.1371/journal.pone.0312197.t003

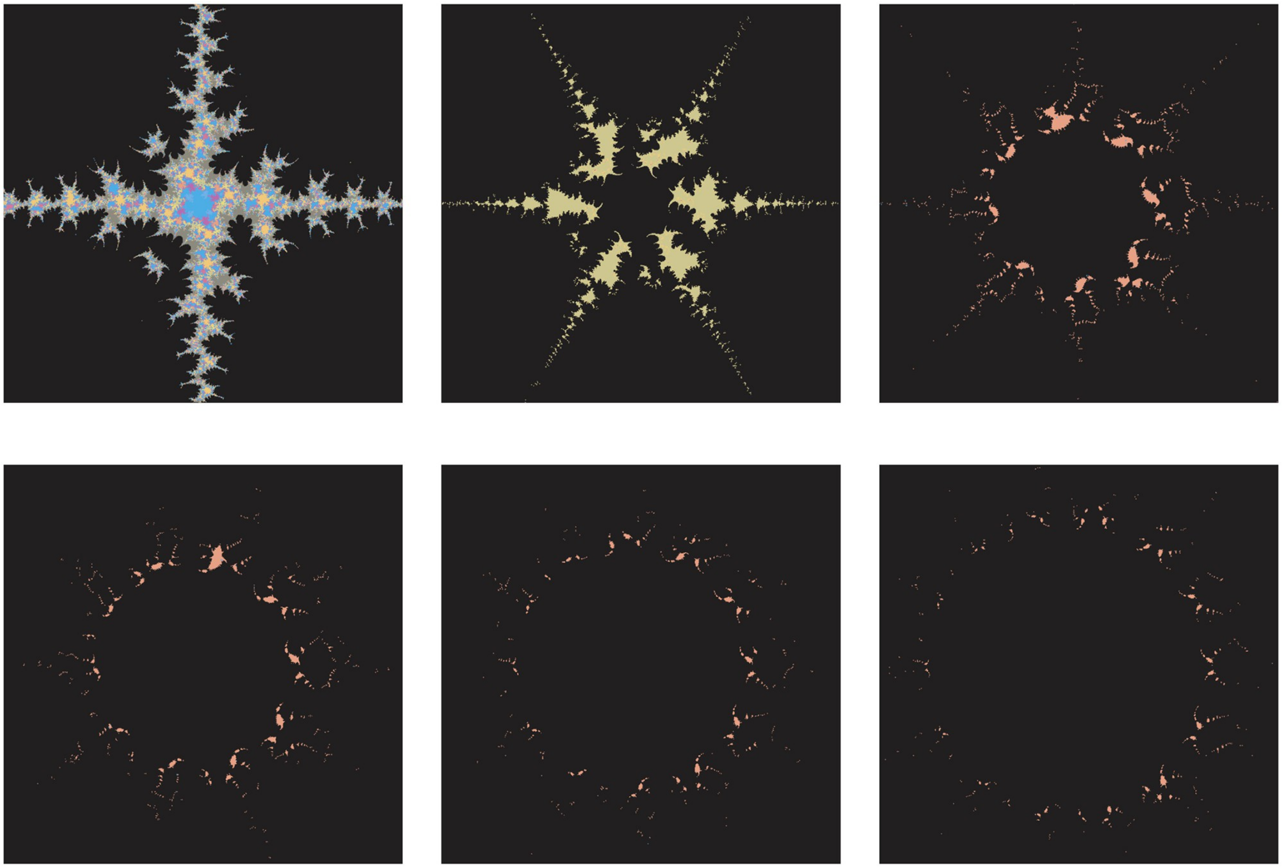

Fig 1. Effect of m on fractals as Julia set.

https://doi.org/10.1371/journal.pone.0312197.g001

Julia set. The number of chaotic small shapes between the attractors in the fractals in Fig 4 increases when the convex parameter s shown in Table 6 is increasing.

There is an alteration in the fundamental shape with an increase in the value of the parameter a. However, the shade of color is lighter for lower values compared to higher values of a in the Julia set. The volume of red color in the middle of the fractals in Fig 5 is decreasing when the parameter a shown in Table 7 is increasing.

There are almost negligible changes in the fractals in Fig 6 with the change of the values of the parameters γ_1, γ_2, γ_3 and γ_4 shown in Table 8.

The significant fractals in Fig 7 result from a random choice of parameters (see Table 9).

4.2 Fractals as Mandelbrot sets

This subsection illustrates the behavior shift in the fractals as Mandelbrot sets for the transcendental cosine function via the AI orbit with s–convexity. Additionally, the fractals alter significantly when even a small modification is made to any of the parameters. Consequently, we modify nearly every parameter and generate the fractals for our orbit, which are shown in the images below (Reading direction: Row by row and Left to right).

The significant fractals that result from varying a parameter m (see Table 10) while holding the other parameters constant are shown in Fig 8. The numbers of chaotic attractors in fractals

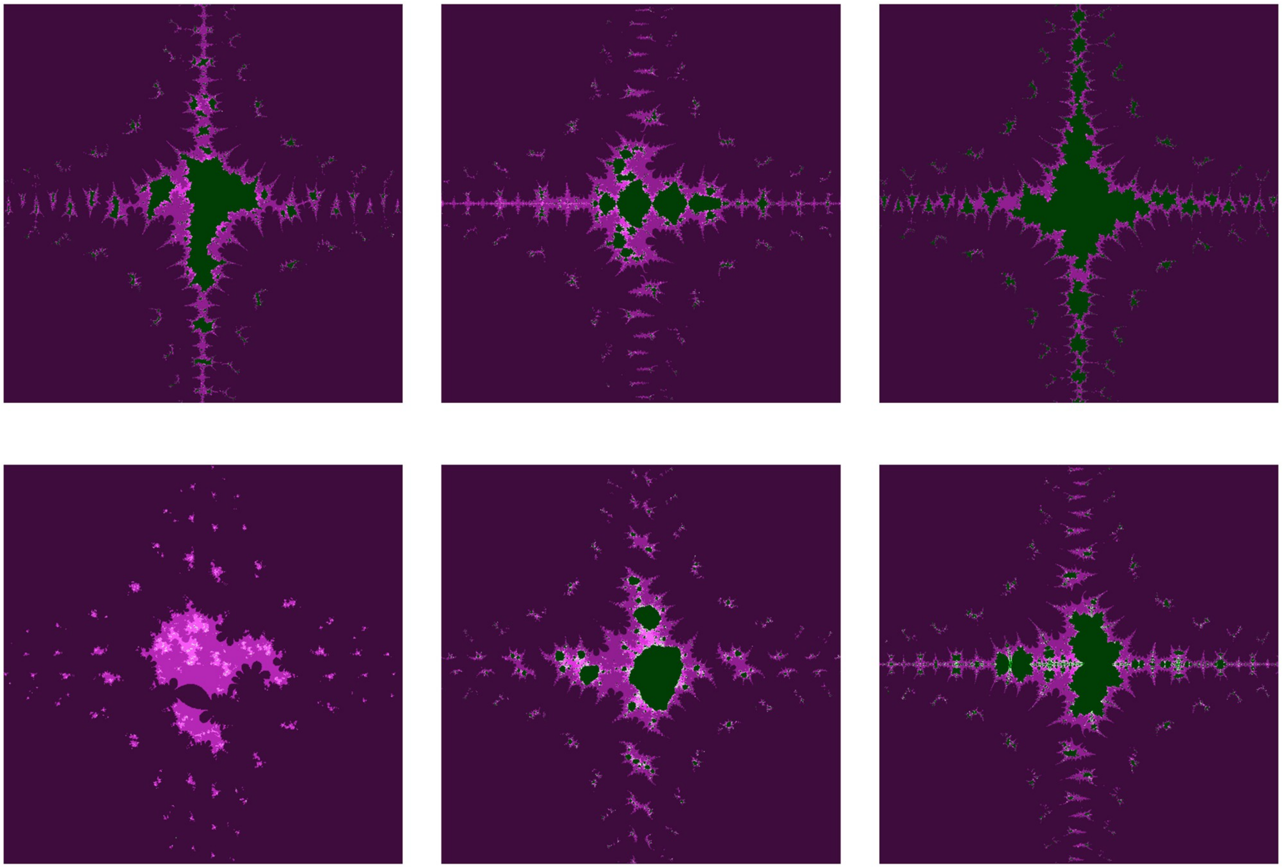

Fig 2. Effect of α on fractals as Julia set.

https://doi.org/10.1371/journal.pone.0312197.g002

are increasing as m increases in number and the number of major blue parts in each Mandelbrot set is $m + 1$ when m is even and m when m is odd.

The fractals are made more beautiful by the parameter α. More aesthetically pleasing fractals can be observed in Fig 9 while maintaining the same values for the other parameters (as in Table 11). The central green portion becomes prominent for complex values of the parameter α.

From the obtained Fig 10 in respect of values in Table 12, we see that a small change of the convex parameter s can be highly effective. A higher value of the convexity parameter enhances

Table 4. Changes in parameter α for generating fractals as Julia set.

	m	α	β	a	s	γ_1	γ_2	γ_3	γ_4
(i)	2	$0.6i$	$-0.085 + 0.004i$	0.003	0.9	0.06	0.08	0.005	0.3
(ii)	2	0.9	$-0.085 + 0.004i$	0.003	0.9	0.06	0.08	0.005	0.3
(iii)	2	$-0.3i$	$-0.085 + 0.004i$	0.003	0.9	0.06	0.08	0.005	0.3
(iv)	2	$0.9 + 0.8i$	$-0.085 + 0.004i$	0.003	0.9	0.06	0.08	0.005	0.3
(v)	2	$-0.6-0.4i$	$-0.085 + 0.004i$	0.003	0.9	0.06	0.08	0.005	0.3
(vi)	2	-0.7	$-0.085 + 0.004i$	0.003	0.9	0.06	0.08	0.005	0.3

https://doi.org/10.1371/journal.pone.0312197.t004

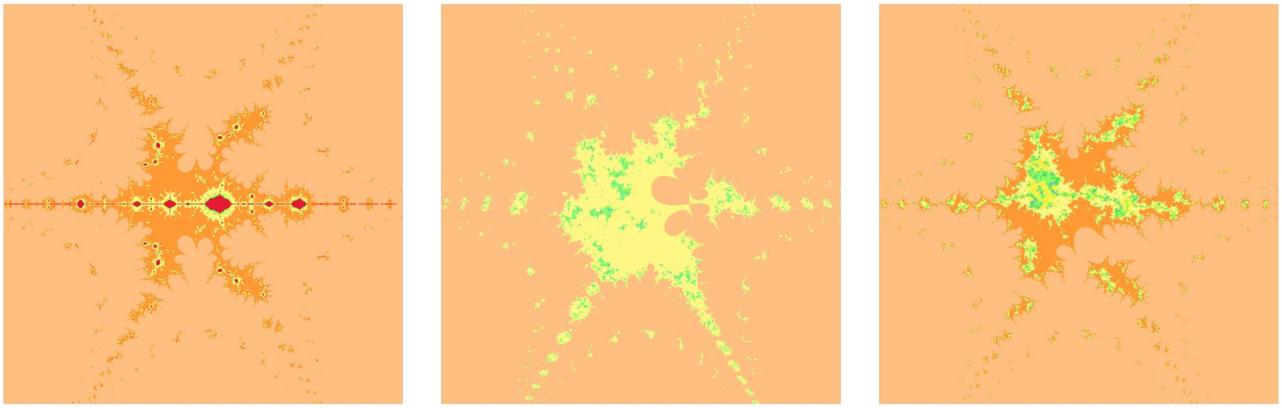

Fig 3. Effect of β on fractals as Julia set.

https://doi.org/10.1371/journal.pone.0312197.g003

Table 5. Changes in parameter β for generating fractals as Julia set.

	m	α	β	a	s	γ_1	γ_2	γ_3	γ_4
(i)	3	0.6	0.3	0.0017	0.987	0.0056	0.0078	0.0095	0.0063
(ii)	3	0.6	$-0.4 + 0.8i$	0.0017	0.987	0.0056	0.0078	0.0095	0.0063
(iii)	3	0.6	$0.06{-}0.12i$	0.0017	0.987	0.0056	0.0078	0.0095	0.0063

https://doi.org/10.1371/journal.pone.0312197.t005

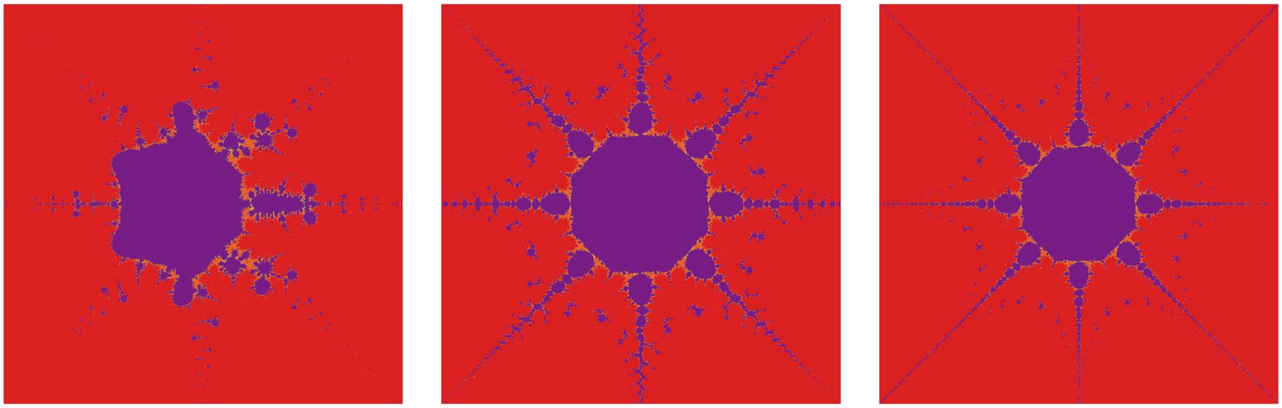

Fig 4. Effect of s on fractals as Julia set.

https://doi.org/10.1371/journal.pone.0312197.g004

Table 6. Changes in parameter s for generating fractals as Julia set.

	m	α	β	a	s	γ_1	γ_2	γ_3	γ_4
(i)	4	−0.0078	$0.006{-}0.0087i$	0.0017	0.16	0.0056	0.0078	0.0095	0.0063
(ii)	4	−0.0078	$0.006{-}0.0087i$	0.0017	0.66	0.0056	0.0078	0.0095	0.0063
(iii)	4	−0.0078	$0.006{-}0.0087i$	0.0017	0.96	0.0056	0.0078	0.0095	0.0063

https://doi.org/10.1371/journal.pone.0312197.t006

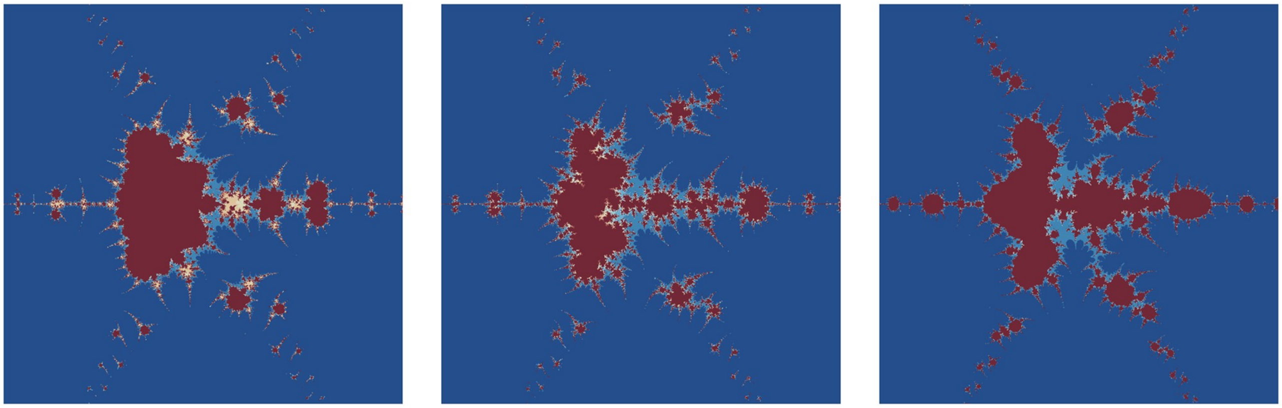

Fig 5. Effect of a on fractals as Julia set.

https://doi.org/10.1371/journal.pone.0312197.g005

Table 7. Changes in parameter a for generating fractals as Julia set.

	m	α	β	a	s	γ_1	γ_2	γ_3	γ_4
(i)	3	0.0078	$0.006 + 0.0087i$	0.17	0.16	0.0056	0.0078	0.0095	0.0063
(ii)	3	0.0078	$0.006 + 0.0087i$	0.67	0.16	0.0056	0.0078	0.0095	0.0063
(iii)	3	0.0078	$0.006 + 0.0087i$	0.97	0.16	0.0056	0.0078	0.0095	0.0063

https://doi.org/10.1371/journal.pone.0312197.t007

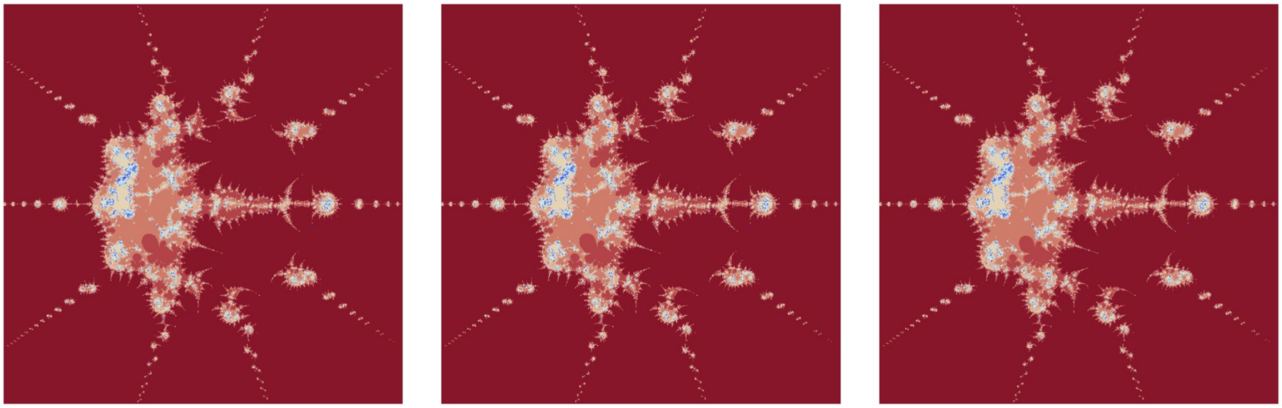

Fig 6. Effect of $\gamma_1, \gamma_2, \gamma_3, \gamma_4$ on fractals as Julia set.

https://doi.org/10.1371/journal.pone.0312197.g006

Table 8. Changes in parameters $\gamma_1, \gamma_2, \gamma_3, \gamma_4$ for generating fractals as Julia set.

	m	α	β	a	s	γ_1	γ_2	γ_3	γ_4
(i)	5	0.078	$0.08 + 0.0087i$	0.67	0.4	0.63	0.009	0.003	0.98
(ii)	5	0.078	$0.08 + 0.0087i$	0.67	0.4	0.006	0.89	0.63	0.008
(iii)	5	0.078	$0.08 + 0.0087i$	0.67	0.4	0.078	0.046	0.023	0.001

https://doi.org/10.1371/journal.pone.0312197.t008

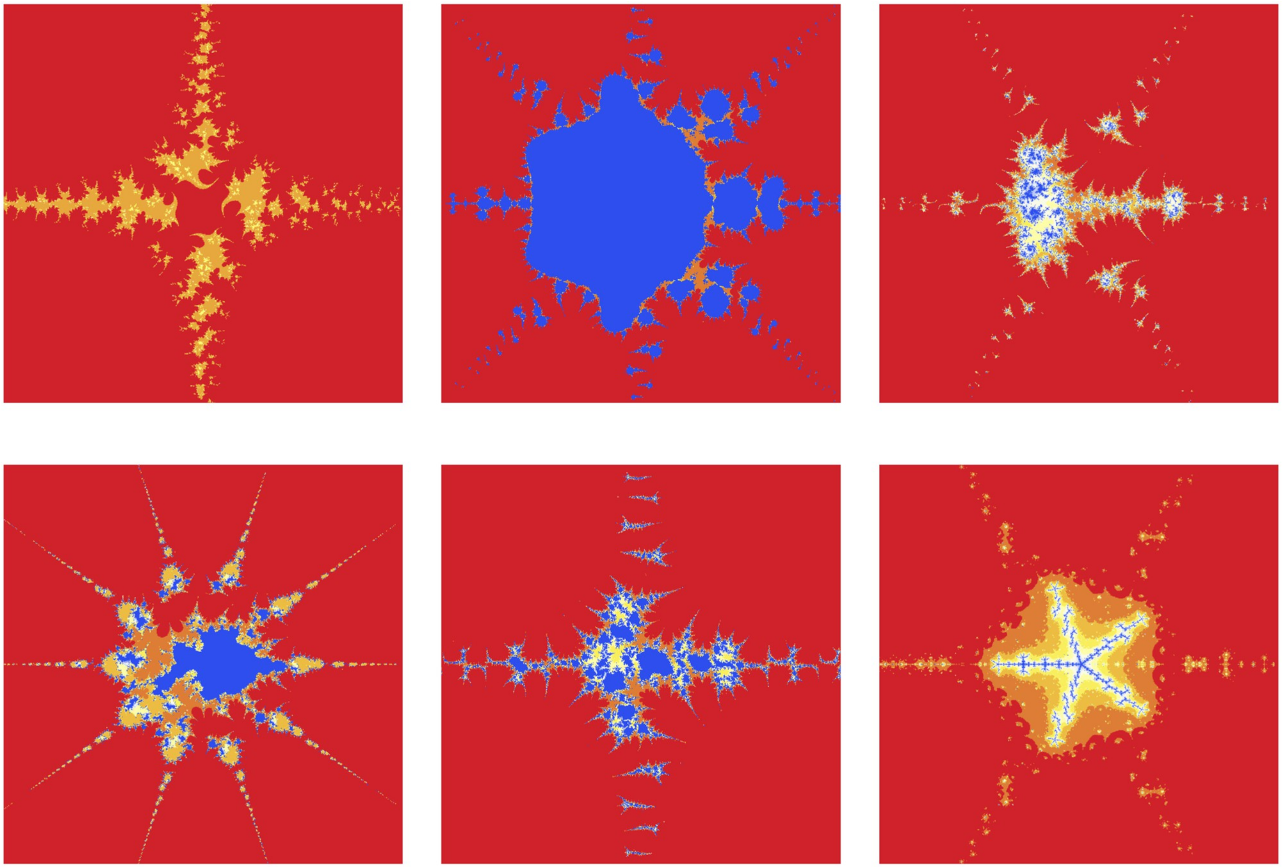

Fig 7. Effect of random choice of parameters on fractals as Julia set.

https://doi.org/10.1371/journal.pone.0312197.g007

the brightness of the Mandelbrot set but decreases the number of colors. A nice fractal having a red color is observed only when $s = 0.1$.

The red color inside the fractals in Fig 11 is decreasing when the parameter a shown in Table 13 is increasing. Also, the fundamental shape changes with an increase in the value of the parameter a.

There are almost negligible changes in the fractals, except for a slight change in the middle in Fig 12 when the parameters γ_1, γ_2, γ_3, γ_4 are changing according to Table 14. Smaller values provide a round shape in the middle by the whitish color.

Table 9. Random changes in parameters for generating fractals as Julia set.

	m	α	β	a	s	γ_1	γ_2	γ_3	γ_4
(i)	2	$0.123i$	$0.251 + i$	0.054	0.934	0.123	0.239	0.563	0.198
(ii)	4	$0.003i$	$0.006i$	0.197	0.734	0.987	0.004	0.207	0.319
(iii)	3	$0.067−0.058i$	$−0.002 + 0.054i$	0.564	0.085	0.284	0.451	0.623	0.746
(iv)	5	$−0.004−0.065i$	$0.00184−0.00156i$	0.981	0.326	0.008	0.002	0.001	0.015
(v)	2	$−0.089 + 0.066i$	0.0253	0.081	0.013	0.574	0.421	0.364	0.298
(vi)	3	−1.823	−0.98	0.375	0.585	0.547	0.755	0.877	0.747

https://doi.org/10.1371/journal.pone.0312197.t009

Table 10. Parameters for generating fractals as Mandelbrot set.

	m	α	a	s	γ_1	γ_2	γ_3	γ_4
(i)	2	$-0.004i$	0.0238	0.7	0.087	0.0932	0.00405	0.6
(ii)	3	$-0.004i$	0.0238	0.7	0.087	0.0932	0.00405	0.6
(iii)	4	$-0.004i$	0.0238	0.7	0.087	0.0932	0.00405	0.6
(iv)	5	$-0.004i$	0.0238	0.7	0.087	0.0932	0.00405	0.6
(v)	6	$-0.004i$	0.0238	0.7	0.087	0.0932	0.00405	0.6
(vi)	7	$-0.004i$	0.0238	0.7	0.087	0.0932	0.00405	0.6

https://doi.org/10.1371/journal.pone.0312197.t010

The significant fractals in Fig 13 result from a random choice of parameters (see Table 15).

Remark 4.1 *It is interesting to notice that generated fractals are highly applicable in the fabric industry, especially in the creation of prints like Batik, Kalamkari, Tie and Dye, and other textile designs(see for instance*, Figs 1, 4–6, 8, 10–13). *They revolutionize textile design by providing endless intricate patterns, automating processes to save time and resources, facilitating scalable designs across different fabric types, and enabling digital previewing to minimize errors and waste. This fosters global collaboration, enhances creativity, reduces costs, and promotes sustainability, ultimately driving market competitiveness and growth in the fabric industry.*

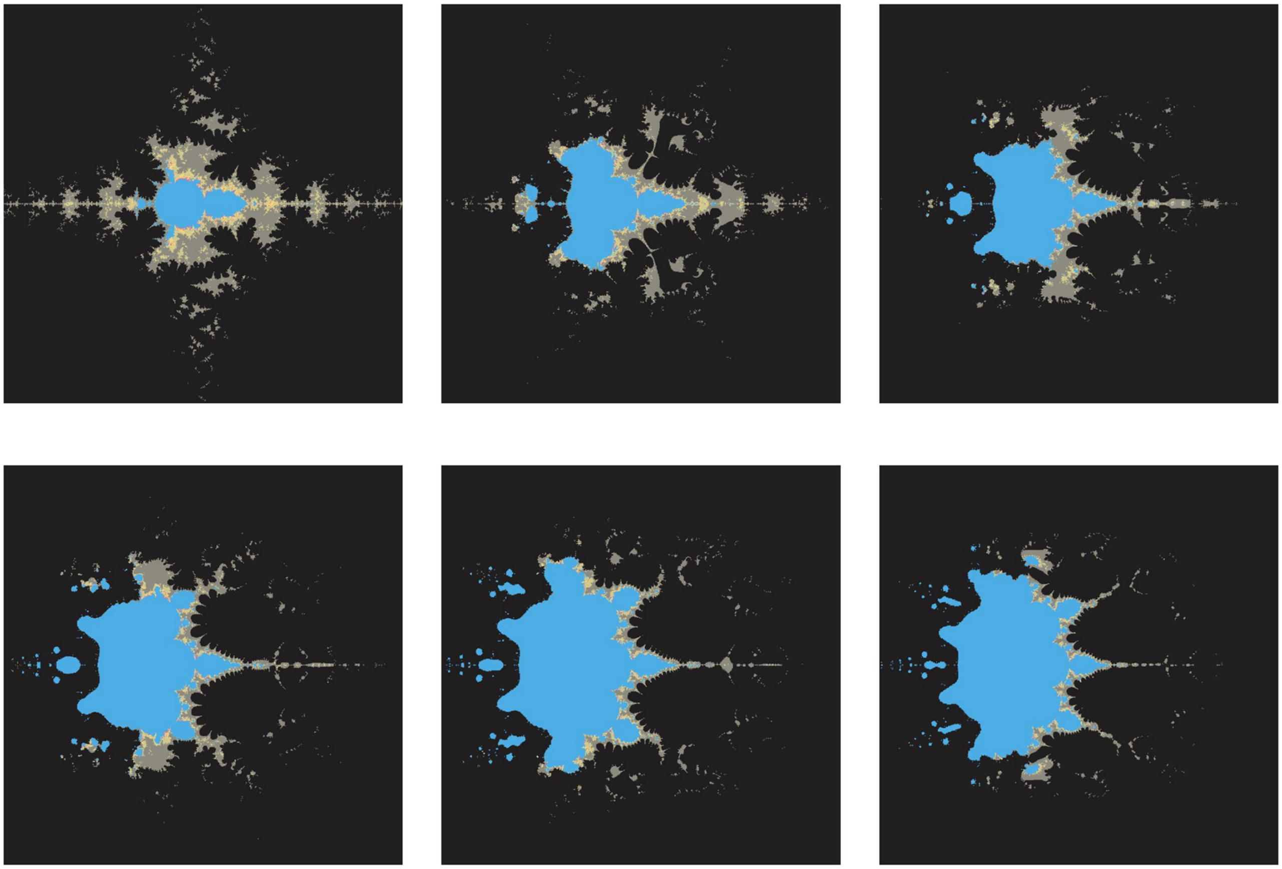

Fig 8. Effect of m on fractals as Mandelbrot set.

https://doi.org/10.1371/journal.pone.0312197.g008

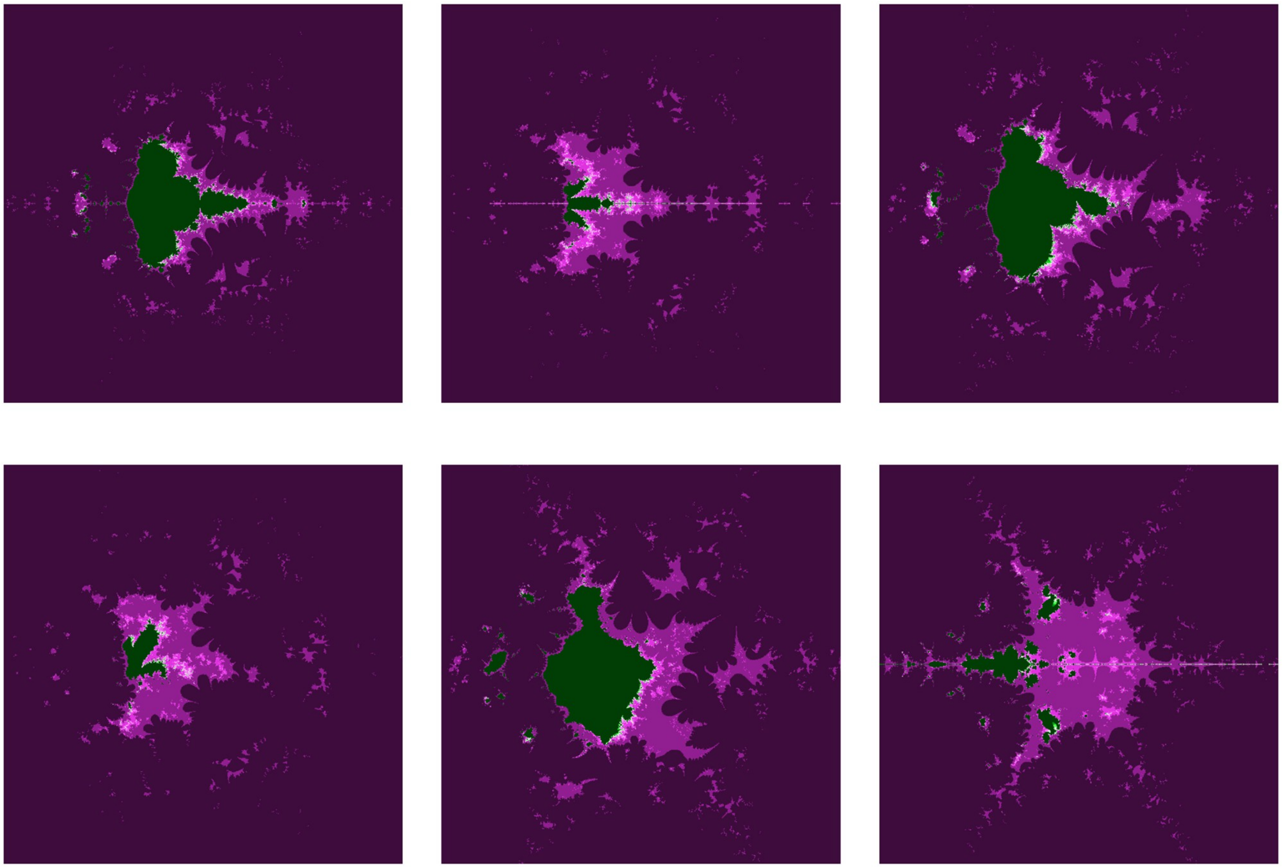

Fig 9. Effect of α on fractals as Mandelbrot set.

https://doi.org/10.1371/journal.pone.0312197.g009

5 Conclusion

Our study presented a novel orbit with s−convexity, and for the subsequent orbit, we generated fractals as Julia and Mandelbrot sets. We provided a result to restrict the escape criterion for transcendental cosine functions of the type $T_{\alpha,\beta}(u) = \cos(u^m) + \alpha u + \beta$, for $u, \alpha, \beta \in \mathbb{C}$ and $m \geq 2$. We also investigated the effects of the relevant parameters on the appearance, dynamics, and color deviation of the formed fractals.

Table 11. Changes in parameter s for generating fractals as Mandelbrot set.

	m	α	a	s	γ_1	γ_2	γ_3	γ_4
(i)	3	$0.5i$	0.013	0.8	0.16	0.09	0.006	0.4
(ii)	3	0.8	0.013	0.8	0.16	0.09	0.006	0.4
(iii)	3	$-0.2i$	0.013	0.8	0.16	0.09	0.006	0.4
(iv)	3	$0.7 + 0.3i$	0.013	0.8	0.16	0.09	0.006	0.4
(v)	3	$-0.3-0.5i$	0.013	0.8	0.16	0.09	0.006	0.4
(vi)	3	-1	0.013	0.8	0.16	0.09	0.006	0.4

https://doi.org/10.1371/journal.pone.0312197.t011

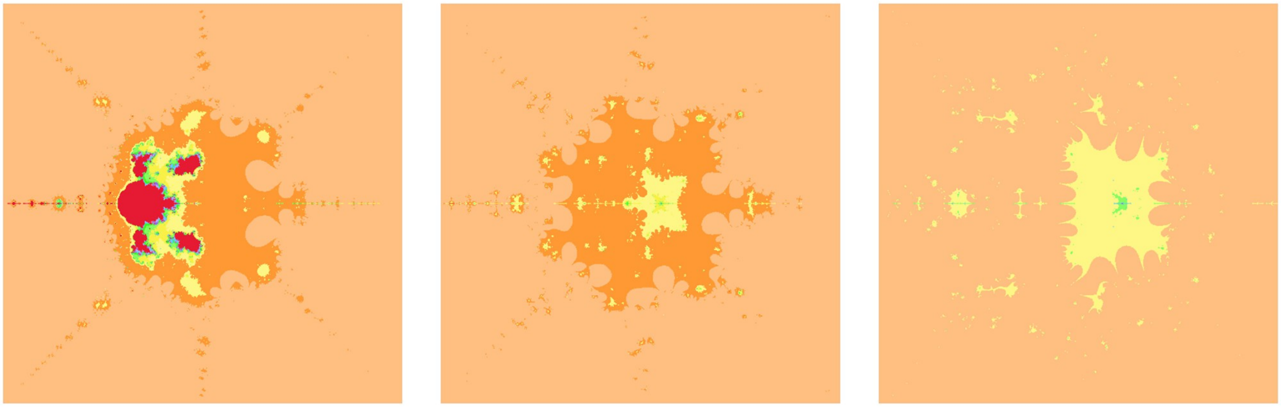

Fig 10. Effect of *s* on fractals as Mandelbrot set.

https://doi.org/10.1371/journal.pone.0312197.g010

Table 12. Changes in parameter s for generating fractals as Mandelbrot set.

	m	α	a	s	γ_1	γ_2	γ_3	γ_4
(i)	4	−2	0.0013	0.1	0.014	0.005	0.003	0.042
(ii)	4	−2	0.0013	0.2	0.014	0.005	0.003	0.042
(iii)	4	−2	0.0013	0.6	0.014	0.005	0.003	0.042

https://doi.org/10.1371/journal.pone.0312197.t012

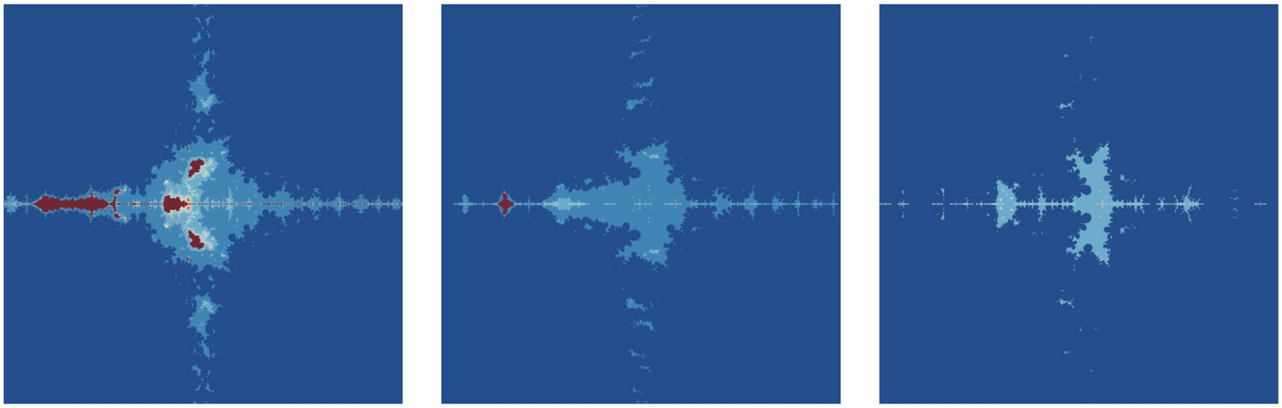

Fig 11. Effect of *a* on fractals as Mandelbrot set.

https://doi.org/10.1371/journal.pone.0312197.g011

Table 13. Changes in parameter a for generating fractals as Mandelbrot set.

	m	α	a	s	γ_1	γ_2	γ_3	γ_4
(i)	2	−2	0.17	0.4	0.014	0.005	0.003	0.042
(ii)	2	−2	0.58	0.4	0.014	0.005	0.003	0.042
(iii)	2	−2	0.99	0.4	0.014	0.005	0.003	0.042

https://doi.org/10.1371/journal.pone.0312197.t013

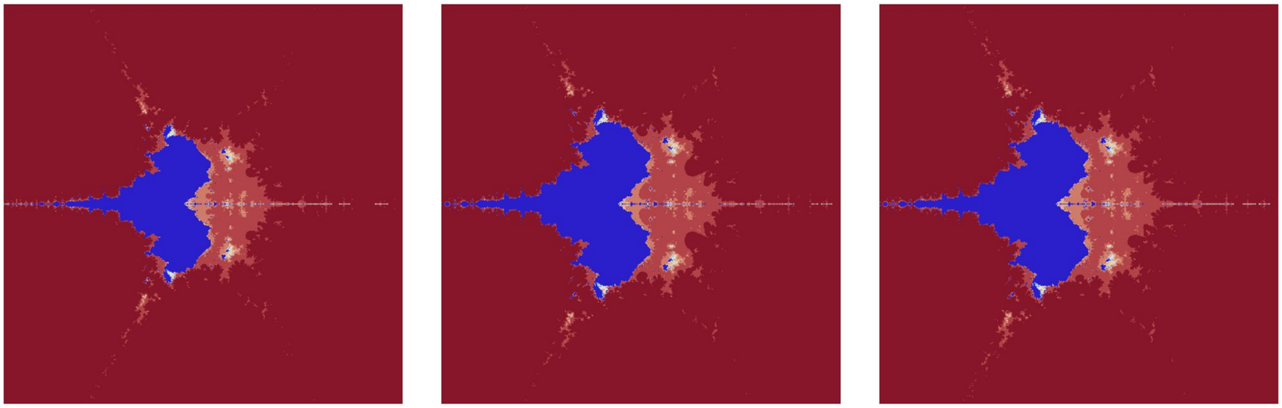

Fig 12. Effect of γ_1, γ_2, γ_3, γ_4 on fractals as Mandelbrot set.

https://doi.org/10.1371/journal.pone.0312197.g012

- It is surprising to see that even small adjustments to one parameter can have a big impact on how the ensuing fractal looks during the generation process, given the same set of values. Therefore, selecting the appropriate parameters is essential to obtaining the intended fractal design.
- In Julia fractals, the number of outer spokes is twice the value of the parameter m, but in Mandelbrot fractals, it is $m + 1$ when m is even and m when m is odd.
- The majority of fractals exhibit symmetry about the initial line.
- In the case of both Julia and Mandelbrot fractals, a small change of the convex parameter s is highly effective.
- Nearly every fractal has a finite number of colors, and every one of them has a hollow section.
- We see that expanding the Mandelbrot set at its petal edges leads to the Julia set, suggesting that each Mandelbrot set point contains a substantial quantity of image data from the Julia set.

The capacity of fractal geometry to capture the complexity of several intricate forms that can be seen in our surroundings is well known. In actuality, however, fractals' chaotic tendencies can represent surfaces and patterns that traditional Euclidean geometry cannot. In the files S1–S4 Figs, we see the beauty of fractals in our mysterious world.

Table 14. Changes in parameter γ_1, γ_2, γ_3, γ_4 for generating fractals as Mandelbrot set.

	m	α	a	s	γ_1	γ_2	γ_3	γ_4
(i)	3	−1	0.8	0.9	0.014	0.005	0.003	0.042
(ii)	3	−1	0.8	0.9	0.175	0.177	0.423	0.568
(iii)	3	−1	0.8	0.9	0.802	0.734	0.608	0.021

https://doi.org/10.1371/journal.pone.0312197.t014

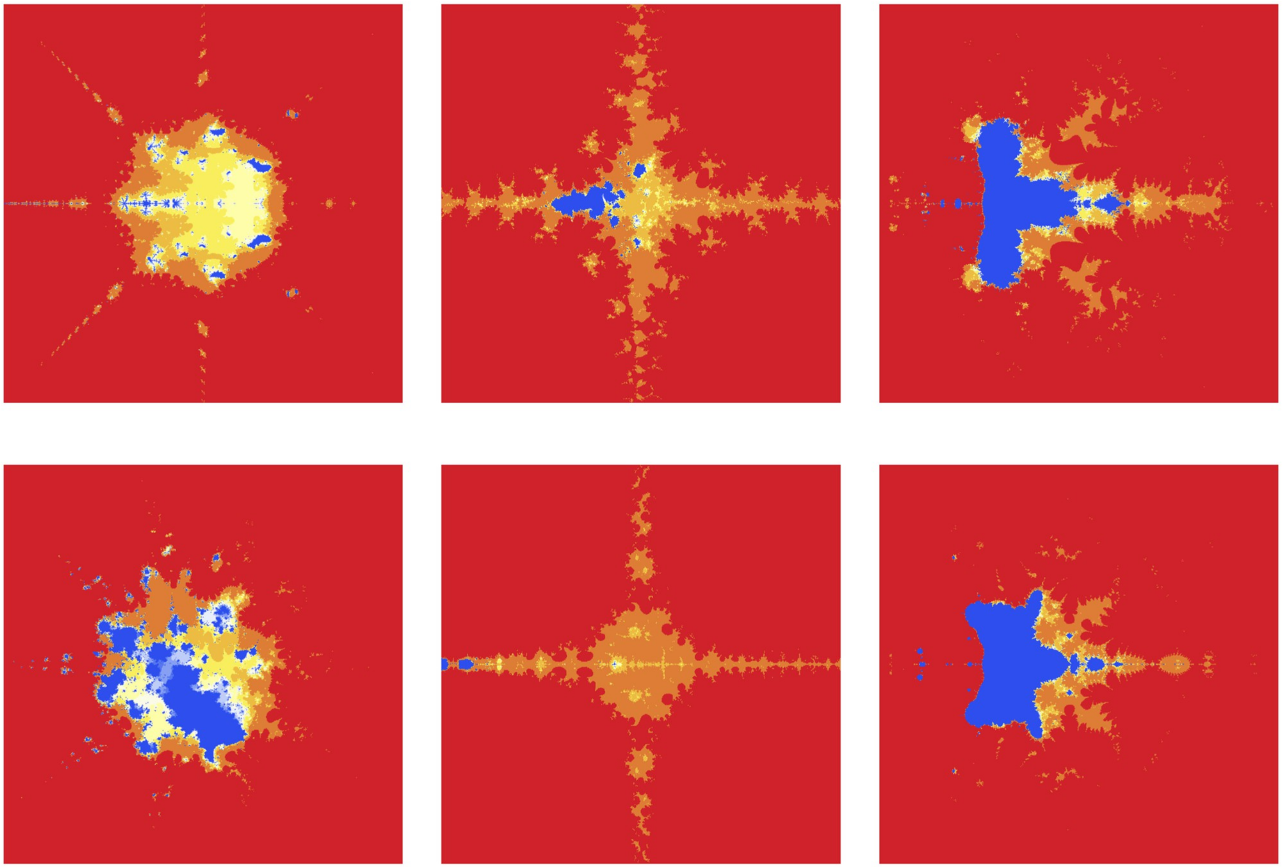

Fig 13. Effect of random choice of parameters on fractals as Mandelbrot set.

https://doi.org/10.1371/journal.pone.0312197.g013

Table 15. Random changes in parameters for generating fractals as Mandelbrot set.

	m	α	a	s	γ_1	γ_2	γ_3	γ_4
(i)	4	−2	0.381	0.943	0.132	0.098	0.046	0.076
(ii)	2	$-1+0.1i$	0.004	0.508	0.654	0.867	0.131	0.312
(iii)	3	$0.2-0.008i$	0.114	0.409	0.534	0.132	0.857	0.973
(iv)	5	$0.9-0.8i$	0.223	0.785	0.705	0.862	0.145	0.213
(v)	2	$-3-0.0061i$	0.004	0.199	0.345	0.892	0.235	0.697
(vi)	4	$-0.001i$	0.001	0.1	0.765	0.098	0.213	0.768

https://doi.org/10.1371/journal.pone.0312197.t015

Supporting information

S1 Fig. Fractals in fabric.
(TIF)

S2 Fig. Fractals in nature.
(TIF)

S3 Fig. Fractals in art and architecture.
(TIF)

S4 Fig. Fractals in human body.
(TIF)

S5 Fig. Julia set source codes.
(TIF)

S6 Fig. Mandelbrot set source codes.
(TIF)

Acknowledgments

The first author expresses gratitude to the University Grants Commission (UGC), New Delhi, India.

Author Contributions

Conceptualization: Khairul Habib Alam, Naeem Saleem.

Formal analysis: Khairul Habib Alam, Yumnam Rohen, Naeem Saleem.

Investigation: Maggie Aphane.

Software: Khairul Habib Alam.

Supervision: Yumnam Rohen.

Writing – original draft: Khairul Habib Alam, Asima Razzaque.

Writing – review & editing: Khairul Habib Alam, Yumnam Rohen, Naeem Saleem, Maggie Aphane.

References

1. Banach S. Sur les operations dans les ensembles abstracts et leur application aux equations integrales. Fund. Math. 1922; 3: 133–181. https://doi.org/10.4064/fm-3-1-133-181
2. Alam KH, Rohen Y, Saleem N. Fixed points of (α, β, F^*) and (α, β, F^{**})-weak Geraghty contractions with an application. Symmetry. 2023; 15(1): 243. https://doi.org/10.3390/sym15010243
3. Alam KH, Rohen Y, Kallel IA, Ahmad J. Solution of an algebraic linear system of equations using fixed point results in C^*-algebra valued extended Branciari S_b-metric spaces. International Journal of Analysis and Applications. 2024; 22: 139. https://doi.org/10.28924/2291-8639-22-2024-139
4. Alam KH, Rohen Y, Tomar A. (α, F)-Geraghty type generalized F-contractions on non-Archimedean fuzzy metric-unlike spaces. Demonstratio Mathematica. 2024; 57(1): 20240046. https://doi.org/10.1515/dema-2024-0046
5. Alam KH, Rohen Y, Tomar A. On fixed point and its application to the spread of infectious diseases model in M_v^b-metric space. Mathematical Methods in the Applied Sciences. 2024; 47(7): 6489–6503. https://doi.org/10.1002/mma.9933
6. Singh MP, Rohen Y, Saleem N, Alam KH, Singh KA, Razzaque A. On fixed point equations involving Geraghty type contractions with solution to integral equation. Mathematics. 2023; 11(24): 4882. https://doi.org/10.3390/math11244882
7. Singh MP, Rohen Y, Alam KH, Ahmad J, Emam W. On fixed point and an application of C^*-algebra valued (α, β)-Bianchini-Grandolfi gauge contractions. AIMS Mathematics. 2024; 9(6): 15172–15189. https://doi.org/10.3934/math.2024736
8. Alam KH, Rohen Y, Tomar A, Sajid M. On fixed points in M_v^b-metric space and solutions to nonlinear matrix equations and differential equations related to beam theory. Journal of Nonlinear and Convex Analysis. 2024; 25(9).

9. Alam KH, Rohen Y, Tomar A, Sajid M. On geometry of fixed figures via φ-interpolative contractions and application of activation functions in neural networks and machine learning models. Ain Shams Engineering Journal. In press.
10. Berinde V. Picard iteration converges faster than Mann iteration for a class of quasi-contractive operators. Fixed Point Theory Appl. 2004; 2: 97–105.
11. Mann WR. Mean value methods in iteration. Proc. Amer. Math. Soc. 1953; 4(3): 506–510. https://doi.org/10.1090/S0002-9939-1953-0054846-3
12. Ishikawa S., Fixed points by a new iteration method, Proc. Amer. Math. Soc. 44(1) (1974), 147–150. https://doi.org/10.1090/S0002-9939-1974-0336469-5
13. Noor MA. New approximation schemes for general variational inequalities. J. Math. Anal. Appl. 2000; 251(1): 217–229. https://doi.org/10.1006/jmaa.2000.7042
14. Alam KH, Rohen Y. An efficient iterative procedure in hyperbolic space and application to non-linear delay integral equation. Journal of Applied Mathematics and Computing. 2024. https://doi.org/10.1007/s12190-024-02134-z
15. Alam KH, Rohen Y, Saleem N, Aphane M, Razzaque A. Convergence of Fibonacci-Ishikawa iteration procedure for monotone asymptotically non-expansive mappings. Journal of Inequalities and Applications. 2024; 2024: 81.
16. Alam KH, Rohen Y. Convergence of a refined iterative method and its application to fractional Volterra-Fredholm integro-differential equations. Computational and Applied Mathematics. 2025; 44(1): 2. https://doi.org/10.1007/s40314-024-02964-4
17. Ofem AE, Igbokwe ID. An efficient Iterative method and its applications to a nonlinear integral equation and delay differential equation in Banach space. Turkis J. Ineq. 2020; 4(2): 79–107.
18. Kang S, Nazeer W, Tanveer M, Shahid A. New fixed point results for fractal generation in Jungck Noor orbit with s-convexity. J. Funct. Spaces 2015; 2015: 963016.
19. Nazeer W, Kang S, Tanveer M, Shahid A. Fixed point results in the generation of Julia and Mandelbrot sets. J. Inequalities Appl. 2015; 2015: 298. https://doi.org/10.1186/s13660-015-0820-3
20. Mishra M, Ojha D, Sharma D. Some common fixed point results in relative superior Julia sets with Ishikawa iteration and s-convexity. Int. J. Adv. Eng. Sci. Technol. 2011; 2(2): 175–180.
21. Cho S, Shahid A, Nazeer W, Kang S. Fixed point results for fractal generation in Noor orbit and s-convexity. Springer Plus 2016; 5: 1843. https://doi.org/10.1186/s40064-016-3530-5
22. Kumari S, Kumari M, Chugh R. Generation of new fractals via SP orbit with s-convexity. Int. J. Eng. Technol. 2017; 9(3): 2491–2504. https://doi.org/10.21817/ijet/2017/v9i3/1709030282
23. Gdawiec K, Shahid A. Fixed point results for the complex fractal generation in the S-iteration orbit with s-convexity. Open J. Math. Sci. 2018; 2(1): 56–72. https://doi.org/10.30538/oms2018.0017
24. Abbas M, Iqbal H, De la Sen M. Generation of Julia and Mandelbrot sets via fixed points. Symmetry 2020; 12(1): 86. https://doi.org/10.3390/sym12010086
25. Ouyang PC, Chung KW, Nicolas A, Gdawiec K. Self-similar fractal drawings inspired by M. C. Escher's print square limit. ACM Trans. Graphic. 2021; 40: 1–34. https://doi.org/10.1145/3456298
26. Martinez F, Manriquez H, Ojeda A, Olea G. Organization patterns of complex river networks in Chile: a fractal morphology. Mathematics 2022; 10: 1806. https://doi.org/10.3390/math10111806
27. Lu X, Sun W. Control and synchronization of Julia sets of discrete fractional Ising models. Chaos, Solitons & Fractals 2024; 180: 114541. https://doi.org/10.1016/j.chaos.2024.114541
28. Orsucci F. Complexity science, living systems, and reflexing interfaces: New models and perspectives, Hershey, PA: IGI Global, 2012.
29. Sreenivasan KR. Fractals and multifractals in fluid turbulence. Annu. Rev. Fluid Mech. 1991; 23: 539–604. https://doi.org/10.1146/annurev.fl.23.010191.002543
30. Kenkel NC, Walker DJ. Fractals in the biological sciences. Coenoses. 1996; 11: 77–100.
31. Devaney RL. A first course in chaotic dynamical systems: theory and experiment, Addison-Wesley, Boston, MA, USA, 2nd edition, 1992.
32. Mandelbrot BB. The fractal geometry of nature, W. H. Freeman, New York, NY, USA, 1982.
33. Barnsley, M. Fractals Everywhere, 2nd ed.; Academic Press: San Diego, CA, USA, 1993.
34. Julia G. Mémoire sur l'itération des fonctions rationnelles. J. Math. Pures Appl. 1918; 8: 47–745.
35. Cohen N. Fractal antenna applications in wireless telecommunications. Prof. Program Proc. Electron. Ind. Forum New Engl. 1997; 43–49. https://doi.org/10.1109/EIF.1997.605374
36. Fisher Y. Fractal image compression. Fractals 1994; 2: 347–361. https://doi.org/10.1142/S0218348X94000442

37. Liu SA, Bai WL, Liu GC, Li WH, Srivastava HM. Parallel fractal compression method for big video data. Complexity 2018; 2018: 2016976. https://doi.org/10.1155/2018/2016976

38. Diao K, Butler D, Ulanicki B. Fractality in water distribution networks: application to criticality analysis and optimal rehabilitation. Urban Water J. 2021; 18: 885–895. https://doi.org/10.1080/1573062X.2021.1948076

39. King CC. Fractal and chaotic dynamics in nervous systems. Prog. Neurobiol. 1991; 36: 279–308. https://doi.org/10.1016/0301-0082(91)90003-J PMID: 1871317

40. Bhoria A, Panwar A, Sajid M. Mandelbrot and Julia sets of transcendental functions using Picard-Thakur iteration. Fractal Fract. 2023; 7: 768. https://doi.org/10.3390/fractalfract7100768

41. Rawat S, Prajapati DJ, Tomar A, Gdawiec K. Generation of Mandelbrot and Julia sets for generalized rational maps using SP-iteration process equipped with s-convexity. Mathematics and Computers in Simulation. 2024; 220: 148–169. https://doi.org/10.1016/j.matcom.2023.12.040

42. Dhurandhar SV, Bhavsar VC, Gujar UG. Analysis of z−plane fractal images from $z \to z\alpha + c$ for $\alpha < 0$. Comput. Graph. 1993; 17: 89–94. https://doi.org/10.1016/0097-8493(93)90056-F

43. Adhikari N, Sintunavarat W. Exploring the Julia and Mandelbrot sets of $z^p + \log c^t$ using a four-step iteration scheme extended with s-convexity. Mathematics and Computers in Simulation 2024; 220: 357–381. https://doi.org/10.1016/j.matcom.2024.01.010

44. Crowe WD, Hasson R, Rippon PJ, Strain-Clark PDE. On the structure of the mandelbar set. Nonlinearity 1989; 2: 541–553. https://doi.org/10.1088/0951-7715/2/4/003

45. Antal S, Tomar A, Prajapati M, Prajapati DJ, Sajid M. Fractals as Julia sets of complex sine function via fixed point iterations. Fractal and Fractional 2021; 5(4). https://doi.org/10.3390/fractalfract5040272

46. Griffin C, Joshi G. Octonionic Julia sets. Chaos Solitons & Fractals 1992; 2: 11–24. https://doi.org/10.1016/0960-0779(92)90044-N

47. Singh S, Jain S, Mishra S. A new approach to superfractals. Chaos Solitons & Fractals 2009; 42(5): 3110–3120. https://doi.org/10.1016/j.chaos.2009.04.052

48. Kumari S, Gdawiec K, Nandal A, Postolache M, Chugh R. A novel approach to generate Mandelbrot sets, Julia sets and biomorphs via viscosity approximation method. Chaos, Solitons & Fractals 2022; 163: 112540. https://doi.org/10.1016/j.chaos.2022.112540

49. Rani M, Kumar V. Superior Julia set. J. Korea Soc. Math. Educ. Ser. D Res. Math. Educ. 2004; 8: 261–277.

50. Chauhan YS, Rana R, Negi A. New Julia sets of Ishikawa iterates. Int. J. Comput. Appl. 2010; 7: 34–42.

51. Li D, Tanveer M, Nazeer W, Guo X. Boundaries of filled Julia sets in generalized Jungck-Mann orbit. IEEE Access 2019; 7: 76859–76867. https://doi.org/10.1109/ACCESS.2019.2920026

52. Kwun YC, Tanveer M, Nazeer W, Abbas M, Kang SM. Fractal generation in modified Jungck–S orbit. IEEE Access 2019; 7: 35060–35071. https://doi.org/10.1109/ACCESS.2019.2904677

53. Antal S, Tomar A, Prajapati DJ, Sajid M. Variants of Julia and Mandelbrot sets as fractals via Jungck-Ishikawa fixed point iteration system with s-convexity. AIMS Math. 2022; 7(6): 10939–10957. https://doi.org/10.3934/math.2022611

54. Pinheiro M. s-convexity: foundations for analysis. Differ. Geom. Dyn. Syst. 2008; 10: 257–262.
